# Rapidly Growing Mycobacteria Associated with Laparoscopic Gastric Banding, Australia, 2005–2011

**DOI:** 10.3201/eid2010.140077

**Published:** 2014-10

**Authors:** Hugh L. Wright, Rachel M. Thomson, Alistair B. Reid, Robyn Carter, Paul B. Bartley, Peter Newton, Christopher Coulter

**Affiliations:** Royal Brisbane and Women’s Hospital, Brisbane, Queensland, Australia (H.L. Wright);; Gallipoli Medical Research Centre, Brisbane (R.M. Thomson);; Wollongong Hospital, Woolongong, New South Wales, Australia (A.B. Reid, P. Newton);; Pathology Queensland, Brisbane (R. Carter, C. Coulter); QML Pathology, Brisbane (P.B. Bartley);; The Prince Charles Hospital, Brisbane (C. Coulter)

**Keywords:** Nontuberculous mycobacteria, gastric banding, rapidly growing mycobacteria, complications bariatric surgery, tuberculosis and other mycobacteria, Australia

## Abstract

Laparoscopic gastric banding is a common bariatric procedure worldwide. Rapidly growing mycobacteria are environmental organisms increasingly seen as pathogens, often in infected prosthetic material. We report 18 cases of infection associated with laparoscopic gastric banding caused by *Mycobacterium fortuitum* and *M. abscessus* in Australia during 2005–2011. We identified cases by reviewing positive cultures at the Queensland state reference laboratory or through correspondence with clinicians, and we obtained clinical and epidemiologic data. Eleven cases of *M. fortuitum* and 7 cases of *M. abscessus* infection were identified. The port was thought to be the primary site of infection in 10 of these cases. Complications included peritonitis, band erosion, and chronic ulceration at the port site. Rapidly growing mycobacteria can infect both port and band and can occur as either an early perioperative or late infection. Combination antimicrobial therapy is used on the basis of in vitro susceptibilities. Device removal seems to be vital to successful therapy.

## Abstract 2

Device removal seems to be vital to successful therapy.

The exponential increase in obesity and morbid obesity worldwide has led to a corresponding increase in bariatric surgical procedures to prevent obesity-associated illness and death (*1*). Laparoscopic gastric banding is a restrictive procedure involving insertion of an inflatable silicon band at the gastric cardia near the gastro-esophageal junction, which enables adjustment of the size of the outlet through the addition or removal of aqueous solution through a subcutaneous port in the abdominal wall. It is the most common bariatric procedure performed in Australia and the United Kingdom (*2*); perceived advantages include its less technical surgical demands and low rates of perioperative complications (*3*). More than 11,000 procedures were performed in Australia during 2011 (*4*). Infection rates are reportedly low (*3*) but can occur at the site of the subcutaneous port or be associated with the band itself.

Rapidly growing mycobacteria are ubiquitous organisms found in environmental sources, including soil and water. They cause skin and soft tissue infections and pulmonary disease but also have a predilection for causing diseases involving implanted prosthetic material. Infections associated with silicone implants, indwelling intravenous or peritoneal catheters, cardiac devices, and prosthetic joints have been reported (*5*–*8*). Isolated cases of mycobacterial infection involving gastric banding have been reported in recent years (*9*,*10*). We report 18 cases of rapidly growing mycobacterial infections associated with laparoscopic gastric banding in Australia during 2005–2011.

## Methods

We identified cases by a variety of methods. We systematically reviewed positive cultures for rapidly growing mycobacteria isolated at Queensland Mycobacterium Reference Laboratory (QMRL, Brisbane, QLD, Australia) that were associated with laparoscopic gastric banding based on the clinical notes provided with the specimen. Other cases were identified by direct clinical involvement by the authors. Additional cases were identified through correspondence with infectious diseases physicians and microbiologists within Australia. Approval was obtained from the local area human research ethics committee associated with the reference laboratory. Clinical and microbiological data for each case, including band manufacturer, technique, and timing of access of the port, as well as the solution used, and treatment records where available were obtained from the treating surgeons, physicians, or microbiologists involved and by examining the hospital medical record.

Organisms were speciated in mycobacterium reference laboratories after referral from laboratories where primary isolation occurred. QMRL characterized 14 isolates using phenotypic and molecular methods, including the Genotype Mycobacteria CM line probe assay (Hain Lifesciences, Nehren, Germany).

Five patients were seen within a narrow temporal period within 9 months of each other, with cultures that isolated *M. fortuitum*. These isolates were further investigated by using pulsed-field gel electrophoresis (PFGE) and repetitive sequence–based PCR (rep-PCR) strain typing using the Diversilab system (bioMérieux, Melbourne, Victoria, Australia) to exclude clonality and delineate the possibility of a point source as the cause of these infections. Because of the wide geographic diversity of cases and delay between infections and outbreak recognition, environmental sampling around the 18 patients was not possible. However, the isolates received were compared with stored environmental isolates from another study (*11*) and other clinical isolates received by QMRL.

For the rep-PCR method, DNA was extracted from 10 clinical isolates by using the Ultraclean Microbial DNA Isolation Kit (MO BIO Laboratories, Carlsbad, CA, USA). The PCR mixture was prepared by using AmpliTaq polymerase and PCR buffer (Applied Biosystems, Hammonton, NJ, USA) and Mycobacterium DiversiLab primer mix according to the manufacturer’s instructions (bioMérieux). Rep-PCR products were separated and detected by microfluidic chips of the Diversilab System. Fingerprints were analyzed with Diversilab software v.3.4.38 by using the Pearson correlation coefficient and unweighted pair group method with arithmetic means to compare isolates and determine clonal relationship. PFGE was performed on the same 10 clinical isolates and results compared with the patterns generated by automated rep-PCR. Based on the Tenover (*12*) classification of isolates using PFGE, the Diversilab rep-PCR similarity cutoffs were determined as >97% (indistinguishable), >95% (similar), and <95% (different).

PFGE was performed by using the method outlined in the BioRad Genpath Group 6 Kit (BioRad, Marnes-la-Coquette, France) with modifications outlined by Mazurek et al. (*13*) and Burki et al. (*14*). Organisms were inoculated into 10 mL Middlebrook 7H9 broth (Difco, Becton Dickinson, Sparks, MD, USA, in-house media) supplemented with 0.2% OADC (Difco, Becton Dickinson), 0.1% Tween 80 (MP Biochemicals, Solon, OH, USA), cycloserine (1 mg/mL; Sigma-Aldrich, St Louis, MO, USA), and ampicillin (0.1 mg/mL; Sigma-Aldrich) and incubated for 3 d. One milliliter of broth was centrifuged and the supernatant discarded.

Gel plugs were prepared and incubated in 500 μL of lysis buffer 1 and 20 μL Lysozyme (25 mg/mL) at 36°C. After a wash step, 500 μL buffer and 20 μL Proteinase K (>600 U/mL) were added to each sample. Plugs were incubated for 48 h at 50°C. The plugs were then washed 4 times in 1× wash buffer. After the final wash, the plugs were stored in 1× wash buffer. Digestion was performed by using *Xba*1 enzyme (10 U/mL), and the samples were incubated for 18 h at 36°C.

The plugs were loaded into wells of a 1% PFGE agarose gel (BioRad), ensuring that no air bubbles formed. Sufficient 0.5× tris-borate-EDTA was added to the PFGE cell and cooled to 14°C, and electrophoresis was performed by using the following parameters: Initial A time 1 s, Final A time 40 s, voltage 200 V, and time 22 h. After electrophoresis, the gel was stained by using ethidium bromide (BioRad), de-stained in running distilled water for 30 min, and then photographed. Antimicrobial susceptibility testing was performed at QMRL by using broth microdilution in accordance with the Clinical and Laboratory Standards Institute guidelines (*15*).

## Results

We identified 18 cases of rapidly growing mycobacterial infections associated with adjustable gastric bands over a 6-year period; the causative organism was *M. fortuitum* in 11 of these patients and *M. abscessus* in 7. Mean age of patients was 45 years; 15 (83%) patients were female (Table 1). The average weight of patients was 133 kg at time of insertion of laparoscopic adjustable gastric band. In 5 patients, diabetes mellitus previously had been diagnosed. No patients had been treated with glucocorticoids or other immunosuppressant medications. Time between initial insertion of device and infection varied widely; 8 (44%) cases occurred within the first 3 months (range 21 days–8 years) after insertion. Ten patients initially had primary port site infection; 3 patients had a concurrent port site infection and abdominal symptoms; and 5 patients had abdominal symptoms alone that suggested primary band infection. The most common symptoms associated with band infection were fever, abdominal pain, nausea, and vomiting. Three patients sought care within 4 weeks after band insertion because of evidence of associated microperforation or erosion of the gastric lumen around the band site endoscopically or intra-operatively. Cultures were often polymicrobial with rapidly growing mycobacteria isolated in the presence of *Staphylococcus* spp., enteric gram-negative organisms, or *Candida albicans*. Of the 8 patients who had primary band involvement or features consistent with combined band/port involvement, 5 sought care within 3 months after insertion.

**Table 1:** Demographic and clinical characteristics of 18 patients with rapidly growing mycobacterial infections complicating laparoscopic gastric band devices, Australia, 2005–2011

Characteristic	Value
Age, mean (range), y	45 (29–64)
M:F	3:15
Weight, mean, kg	133
Co-morbidities, no. (%)	
Diabetes mellitus	5 (28)
Hypertension	6 (33)
Obstructive sleep apnea	4 (22)
Depression	4 (22)
Immunosuppression/glucocorticoid use	0
Causative organism, no. (%)	
* M. fortuitum*	11 (61)
* M. abscessus*	7 (39)
Primary site of infection, no. (%)	
Port	10 (56)
Band	5 (28)
Combined port/band	3 (16)
Time from insertion to presentation, no. (%)	
Early, <3 mo	8 (44)
Late, >3 mo	10 (56)
Associated complications, no.	
Peritonitis	2
Erosion/perforation	5
Chronic ulcer	2
Antimicrobial drug therapy	
Median duration (range), mo	6 (3–12)
Combination therapy, no. (%)	17 (94)

Patients who had infection at the primary port site commonly had more indolent signs and symptoms. Pain and erythema at the site were commonly reported. Most of these patients received initial empiric therapy for common bacterial skin and soft tissue pathogens before the causative organism was identified.

Complications included granulomatous peritonitis in 2 patients for whom *M. abscessus* was confirmed on peritoneal biopsy. Infection associated with erosion at the band site occurring >1 month after insertion occurred in 2 additional cases. In 3 patients with primary port site infections, chronic ulcers developed at the port site after device removal; mean time to resolution of ulcer was 9 months, and *M. abscessus* was the causative agent in 2 of these cases.

**Figure 1:**
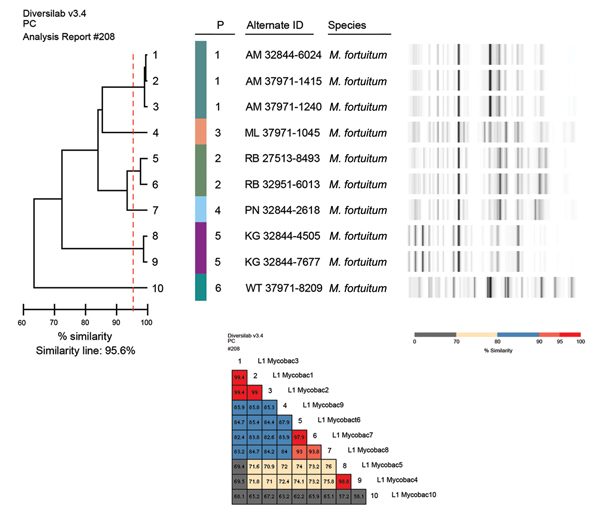
Strain typing using Diversilab platform (bioMérieux, Melbourne, Victoria, Australia) and pulsed-field gel electrophoresis of *Mycobacterium fortuitum* isolates.

PFGE and strain typing by using Diversilab platform on 5 *M. fortuitum* isolates showed sufficient genetic diversity to exclude clonality (Figure 1). Gastric bands in this series were inserted atdifferent centers; given the temporal and geographic diversity of cases, no single point source was identified. In cases where the ports had been accessed, all clinicians reported use of sterile saline or sterile water as the solution for band inflation, performed using sterile techniques by the surgeons themselves.

**Figure 2:**
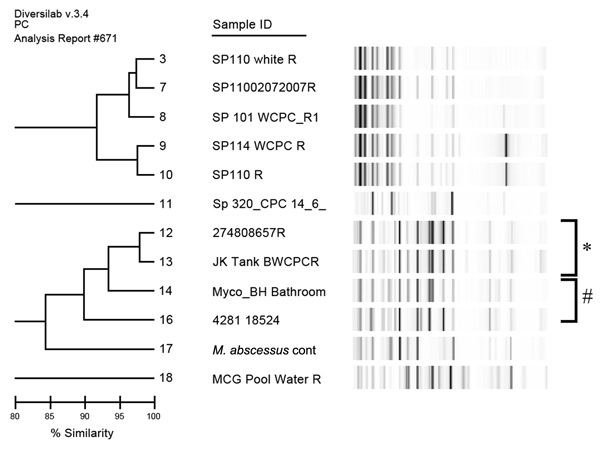
Repetitive sequence–based PCR dendrogram comparing strain types of 2 *Mycobacterium abscessus* isolates associated with laparoscopic band infections, with a laboratory control strain, and 9 other environmental isolates, Australia. *Isolate 12 (patient PB) is indistinguishable from strain 13, isolated from a domestic rainwater tank. #Strain 16 (patient MC) shares 90% similarity with an epidemiologically unrelated domestic bathroom water isolate.

**Figure 3:**
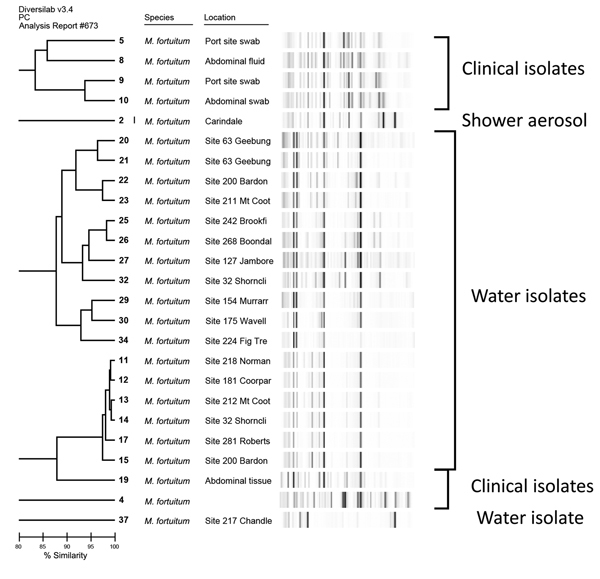
Repetitive sequence–based PCR dendrogram demonstrating differences between *Mycobacterium fortuitum* isolates associated with lap band infections and *M. fortuitum* isolated from water samples. Scale bar indicates % similarity. Source: DiversiLab v. 3.4 PC #675.

**Figure 4:**
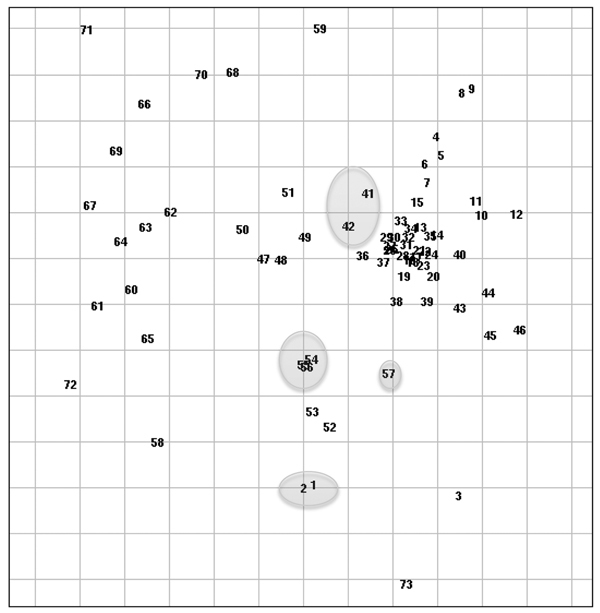
Repetitive sequence–based PCR scatterplot demonstrating lap band isolates (circled) relative to other clinical strains of *Mycobacterium fortuitum* associated with community-acquired and nosocomial infections. Spacing between samples may be distorted if the dataset is large and/or if there is no distinct clustering. Gridline spacing: 5% similarity. Source: DiversiLab v. 3.4 PC #675 (bioMérieux, Melbourne, Victoria, Australia).

The strain of *M. abscessus* isolated from 1 of the patients with primary band infection was indistinguishable from an environmental isolate recovered from a suburban rainwater tank. Another patient with *M. abscessus* infection had a strain that differed from the environmental water isolates recovered from another study (Figure 2). The *M. fortuitum* isolates that were available for strain typing differed from those isolated from municipal water (Figure 3) and from other clinical isolates associated with both community and nosocomial infections (Figure 4).

No clear temporal relationship was identified between access of the port and development of port site infection. No cases were reported to have developed within 4 weeks after most recent access. In at least 4 cases, time from most recent access to development of symptoms at port site was >1 year.

All patients received empiric antibacterial therapy before isolation of the mycobacterium. In 1 case of granulomatous peritonitis, empiric first-line therapy for tuberculosis was begun pending identification of the causative organism. Seventeen patients initially received combination therapy upon confirmation of growth of rapidly growing mycobacteria. Initial intravenous therapy was administered in 5 of the 11 cases involving *M. fortuitum* and all **7** involving *M. abscessus*. Duration of intravenous therapy ranged from 2 to 6 weeks and was followed by ongoing oral therapy. The agents used intravenously were amikacin (12 cases), cefoxitin (10 cases), and imipenem (2 cases). Combination therapy with ciprofloxacin and trimethoprim/sulfamethoxazole was the most common regimen used for *M. fortuitum* infections (7 of 11 cases) and reflected the susceptibility data for the isolates (Table 2). Other agents used were clarithromycin, doxycycline, and minocycline. Therapy for the **7** patients with *M. abscessus* infections was more uniform; oral clarithromycin was used in all cases after an initial intensive phase of amikacin and cefoxitin. Total duration of antimicrobial therapy ranged from 3 to 12 months (median 6 months).

**Table 2:** Susceptibilities of *Mycobacterium* spp. isolated in laparoscopic gastric banding to selected antimicrobial agents, Australia, 2005–2011

Antimicrobial agent	*M. fortuitum*, no. (%), n = 11	*M. abscessus*, no. (%), n = 7
Amikacin	10 (91)	7 (100)
Cefoxitin	7 (64)	5 (71)
Clarithromycin	3 (27)	7 (100)
Ciprofloxacin	10 (91)	0
Minocycline	2 (18)	0
Imipenem	8 (80)	0
Trimethoprim/sulfamethoxozole	9 (82)	0
Amoxycillin/clavulanate	1 (13)	0

In all cases, infection was cured only with complete explantation of the device. In 5 cases for which initial signs and symptoms were consistent with primary port infection, an initial strategy of conservation of the band component was attempted in conjunction with antimycobacterial therapy. In all instances, symptoms subsequently recurred, which resulted in the need for complete removal of the device.

## Discussion

Rapidly growing mycobacteria are increasingly recognized as major pathogens, capable of causing a wide spectrum of clinical illness (*16*). Infection from these organisms after surgical procedures, although uncommon, has been well described and is often seen when the procedure involves implantation of prosthetic material (*17*). The mode of acquisition of infection remains unclear in some cases. In cases that occurred shortly after surgery, infection is likely to have been acquired at the time of surgery. *M. fortuitum* and *M. abscessus* have been reported as causes of wound infections from a variety of surgical procedures, including contamination of aqueous solutions or of the surgical equipment used (*18*,*19*).

Minor trauma has been reported as a risk factor for rapidly growing mycobacterial skin and soft tissue infections (*20*). An advantage of laparoscopic gastric banding is the ability to inflate or deflate the band to alter its restrictive effect; however, this procedure might provide a possible portal of entry for infection, particularly in the absence of strict aseptic technique. Alternatively, mycobacterial colonization of the solution used to inflate or deflate the band could result in infection (*21*). We did not find any history of antecedent injury before port site infection, and accessing the port to adjust the band was not associated temporally with port site infection. Although the port and band is a contiguous device, and infection with 1 component appears to lead to involvement of the entire device, the possible pathogenesis of infection might differ depending on the anatomic site at which infection develops primarily. Most cases in which the band was primarily involved were associated with injury to the gastric wall: microperforation or erosion occurred in 5 (63%) of 8 patients. Infection might have been a secondary event that occurred after perforation and subsequent contamination of the band with gastric contents. Alternatively, band infection itself might have factored in damaging gastric integrity.

Devices or implants can become colonized during manufacture because mycobacteria are present within the environment, especially in water sources. Implantation of colonized porcine heart valves has resulted in pericarditis and endocarditis (*22*). We found no evidence to suggest that any cases in our current report resulted from such colonization; devices from different manufacturers were used and the cases were sporadic. Further investigation of cases in which presentation (though not insertion of the gastric band) was temporally related, showed that the isolates differed enough to exclude a point source.

Trial data to inform treatment of rapidly growing mycobacteria are lacking. In vitro susceptibilities vary between species, although resistance to first-line antituberculosis agents is common in *M.* *fortuitum* and *M. abscessus*. Current guidelines from the American Thoracic Society and the Infectious Diseases Society of America (ATS/IDSA) (*23*) suggest therapy on the basis of susceptibility testing and advocate combination therapy. Macrolides are commonly used and are often the only freely available oral agent with activity against *M. abscessus*, as shown in our report. However, rapidly growing mycobacteria can develop resistance by mutations in the peptidyltransferase region of the 23S ribosome gene (*24*). Furthermore, inducible macrolide resistance has been demonstrated in *M. fortuitum* (*25*) and *M. abscessus* (*26*); thus, monotherapy with this agent is not recommended, even if the isolate appears susceptible. The ATS/IDSA guidelines suggested that treatment for serious soft tissue infections caused by *M. abscessus* consists of clarithromycin, with initial therapy also including amikacin with or without cefoxitin. Suggested treatment for *M. fortuitum* infection is combination therapy with at least 2 active agents as guided by in vitro susceptibilities to prevent development of resistance (*27*). Treatment of the infections reported here is consistent with these guidelines. For infected prosthetic material, as shown here, removal of such material appears to be a critical factor in treatment success and is strongly recommended (*23*). Optimal duration of adjuvant antimicrobial therapy remains elusive and may be influenced by how promptly the device is removed.

Laparoscopic gastric banding is a safe and effective method to enable weight loss in obese patients. It remains the most common bariatric surgery performed in Australia; perioperative death rates are very low (*28*). However, evidence is mounting of increasing rates of long-term complications associated with gastric banding in comparison with the other common bariatric procedure performed worldwide, Roux-en-Y gastric bypass (*29*). Late complications reported include higher rates of long-term reoperation, band slippage with pouch dilation, port dislocation, erosions, and infection of port or band. Evidence also exists that roux-en-Y gastric bypass provides greater excess weight loss (*30*), with a greater reduction of obesity-associated co-morbid conditions (*31*).

Our retrospective series has limitations. Case finding relied in part on recollection of the physician or surgeons interviewed. We reviewed positive cultures from the reference laboratory where the rapidly growing mycobacteria were isolated , but because review relied on adequate clinical notes to identify cases associated with gastric banding, some cases might have been missed. We included cases that occurred early and late after device implantation, which might encompass several different etiologic processes. Although we conducted both epidemiologic and molecular investigations, a clear source of infection was not identified. The treatment observed was not standardized as may be attempted in a prospective trial, which may give clearer guidance as to optimal approach.

*M. fortuitum* and *M. abscessus* should be considered as possible etiologic agents of infection associated with laparoscopic gastric banding, arising from port or band. Infection can occur early during the perioperative period or many years after insertion. Prolonged therapy with combination antimicrobial agents is suggested in conjunction with complete removal of the device.
